# Effect of mechanical stimulation on tissue heterotopic ossification: an *in vivo* experimental study

**DOI:** 10.3389/fphys.2023.1225898

**Published:** 2023-10-11

**Authors:** Zhengya Zhu, Zhongyuan He, Tao Tang, Fuan Wang, Hongkun Chen, Jiaxiang Zhou, Chengkai Lin, Guoliang Chen, Jianmin Wang, Jianfeng Li, Xizhe Liu, Zhiyu Zhou, Shaoyu Liu

**Affiliations:** ^1^ Innovation Platform of Regeneration and Repair of Spinal Cord and Nerve Injury, Department of Orthopaedic Surgery, The Seventh Affiliated Hospital, Sun Yat-sen University, Shenzhen, China; ^2^ Department of Orthopaedic Surgery, The Affiliated Hospital of Xuzhou Medical University, Xuzhou, China; ^3^ Guangdong Provincial Key Laboratory of Orthopedics and Traumatology, Department of Spinal Surgery, Orthopaedic Research Institute, The First Affiliated Hospital of Sun Yat-sen University, Guangzhou, China; ^4^ Department of Orthopaedic Surgery, The Affiliated Hospital of Qingdao University, Qingdao, China; ^5^ Department of Orthopaedic Surgery, The First Affiliated Hospital, Jinan University, Guangzhou, China

**Keywords:** animal model, chronic spinal cord injury, external fixation, ossification of the posterior longitudinal ligament (OPLL), treadmill training, yes-associated protein

## Abstract

**Background:** Heterotopic ossification of tendons and ligaments (HOTL) is a common clinical condition characterized by the absence of discernible features and a lack of effective treatment. *In vitro* experiments have demonstrated that mechanical stimulation can induce cell differentiation toward osteogenesis, thereby promoting heterotopic ossification. Currently, there are few experimental designs aimed at inducing ligament stretching in mice, and the mechanism of heterotopic ossification may not entirely mirror that observed in clinical cases. Therefore, there is an urgent imperative to develop a novel and feasible animal model.

**Methods:** In this study, all the *Enpp1* gene deficiency mice (a mouse model with heterotopic ossification of multiple ligaments) were divided into three groups: the control group, the spinal brake group, and the hyperactive group (treadmill training group). An external spinal fixation device was designed to restrict mice’s spinal flexion and extension at 6 weeks of age. The brace was adjusted weekly according to the changes in the size of the mice. Additionally, treadmill training was used to increase activity in the spinal ligaments and Achilles tendons of the mice. Micro-CT scanning and HE staining were performed at 12, 20, and 28 W to evaluate the degree of ossification in the spinal ligament and Achilles tendon. What’s more, As one of the mechanical stimulation transduction signals, YAP plays a crucial role in promoting osteogenic differentiation of cells. Immunofluorescence was utilized to assess YAP expression levels for the purpose of determining the extent of mechanical stimulation in tissues.

**Results:** Our findings showed that a few ossification lesions were detected behind the vertebral space of mice at 8 weeks of age. Spinal immobilization effectively restricts the flexion and extension of cervical and thoracic vertebrae in mice, delaying spinal ligament ossification and reducing chronic secondary spinal cord injury. Running exercises not only enhance the ossification area of the posterior longitudinal ligament (PLL) and Achilles tendons but also exacerbate secondary spinal cord injury. Further immunofluorescence results revealed a notable increase in YAP expression levels in tissues with severe ossification, suggesting that these tissues may be subjected to higher mechanical stimulation.

**Conclusion:** Mechanical stimulation plays a pivotal role in the process of heterotopic ossification in tissues. Our study provided valid animal models to further explore the pathological mechanism of mechanical stimulation in HOTL development.

## 1 Introduction

Heterotopic ossification of tendons and ligaments (HOTL) is a chronic and disabling disease that causes significant damage to patients’ health and economic burden to society. Currently, there are several known causes of HOTL, including genetic factors, tissue injury, abnormal mechanical stress, age, and metabolic diseases (Biz et al., 2015; Zhang et al., 2020). As a controllable factor, mechanical stress is a topic of interest in research. *In vitro* experimental results demonstrated that primary cells derived from patients with heterotopic ossification exhibit significantly elevated expression of osteogenic marker genes under dynamic culture conditions compared to static cultures, suggesting that tensile stimulation may enhance the osteogenic differentiation of ligaments (Katsumi et al., 2016; Kang et al., 2019; Huber et al., 2020). Moreover, clinical reports suggest that cervical laminoplasty combined with instrumented fusion is more effective in suppressing the progression of the ossification of the posterior longitudinal ligament (OPLL) than stand-alone laminoplasty (Katsumi et al., 2016; Lee et al., 2017; Kang et al., 2019). *In vivo* experiments in animal models have shown that repeated excessive traction stimulation of the rat tail or thoracic vertebra can induce heterotopic ossification of local ligaments (Tsukamoto et al., 2006; Zhao et al., 2021). Given that internal spinal fixation can effectively delay the progression of OPLL, it is essential to construct a model that limits animal physical activity to study specific physiological mechanisms.

Additionally, there is an ongoing debate regarding the role of physical activity in the development of heterotopic ossification. Some scholars assert that passive physical activity can effectively delay traumatic heterotopic ossification and increase joint mobility (Vasileiadis et al., 2021; Vasileiadis et al., 2023). Conversely, other researchers suggest that movement of the spine or Achilles tendon may actually enlarge the ossification range and severely impact the patient’s quality of life (Kang et al., 2019). It is important to note that mechanical stimulation may have additional effects on secondary chronic spinal cord injury (SCI) caused by spinal stenosis beyond affecting the size of the ossification range in OPLL. However, there is no consensus on the role of proper running exercise in OPLL-related secondary chronic spinal cord injury. Takahiro argued that running exercise is an important rehabilitation measure for spinal cord function recovery, as proper exercise can not only promote spinal cord recovery but also improve motor function (Shibata et al., 2021). However, secondary SCI caused by OPLL differs fundamentally from traumatic SCI, and exercise cannot relieve spinal cord compression secondary to spinal stenosis. In contrast, excessive bending of the spine may promote repeated friction stimulation between the spinal cord and the ossification site, and a large number of inflammatory factors may further damage spinal cord tissue.

Our previous study demonstrated the significance of yes-associated protein (YAP), a transcriptional coactivator that plays a crucial role in promoting osteogenic differentiation of cells under tensile stress (Zhu et al., 2023). However, its *in vivo* relevance has yet to be validated. Therefore, we generated a mouse model with *Enpp1* gene deletion that develops spontaneous heterotopic ossification in tendons and ligaments. Our objective was to assess abnormal YAP activation by identifying the ossification focus, which is essential for our subsequent pathological mechanism research.

**FIGURE 1:**
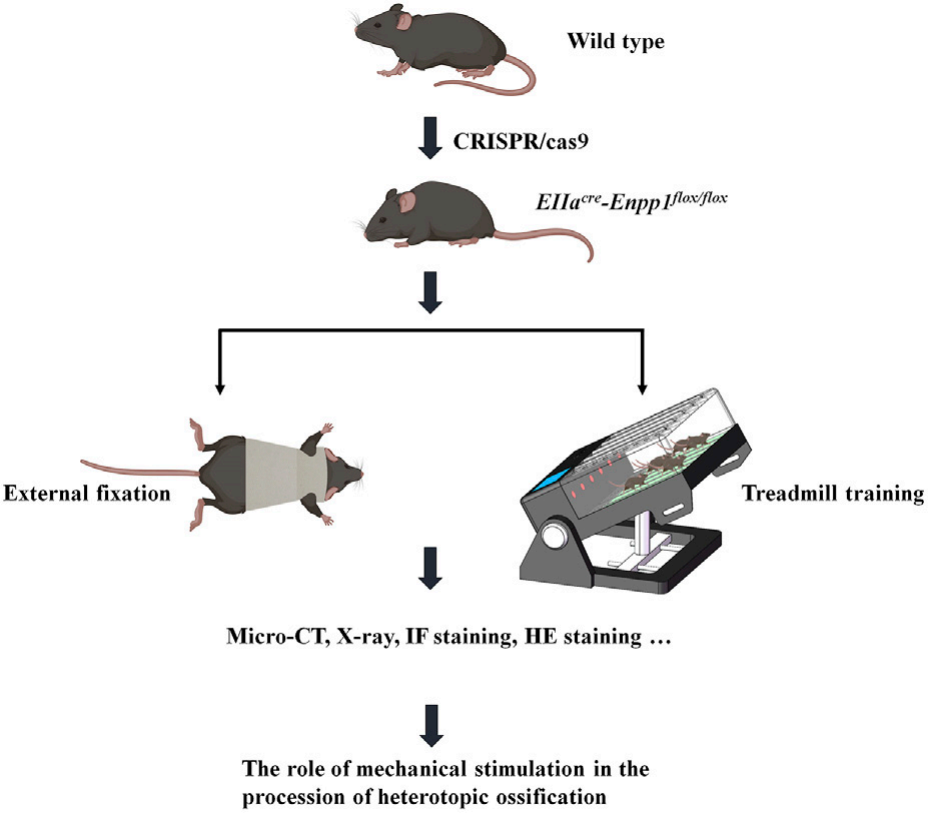
Flow chart.

In this study, we utilized this gene-deficient mouse model to develop an external fixation brace that restricts the spine’s flexion and extension and evaluated its effect on delaying ossification progression. Similarly, a sloped treadmill was utilized to induce spine and Achilles tendon activity, allowing us to observe the ossification range and gait changes (Figure 1). Through this experimental design, we aimed to explore the effect of mechanical stimulation on heterotopic ossification *in vivo*.

## 2 Materials and methods

### 2.1 Animals and diet

The transgenic mice used in this study were raised indoors and provided with a normal laboratory diet. All behavioral experiments were performed during the same circadian cycle (with natural light from 8:00 a.m. to 8:00 p.m.). This animal study was conducted in accordance with ethical guidelines and was approved by the Sun Yat-sen University Animal Care and Use Committee (approval no. SYSU-IACUC-2020-000541).

### 2.2 Mouse genotype identification

Mouse genotyping was conducted using standard protocols. Briefly, 2-mm tail fragments were clipped from 2-week-old mice, and the DNA was extracted and amplified using PCR. The genotype of the mice was determined by analyzing the results of PCR amplification using a DNA gel assay (detailed protocol provided in Supplementary Material S1).

### 2.3 Spinal external fixation device

**FIGURE 2:**
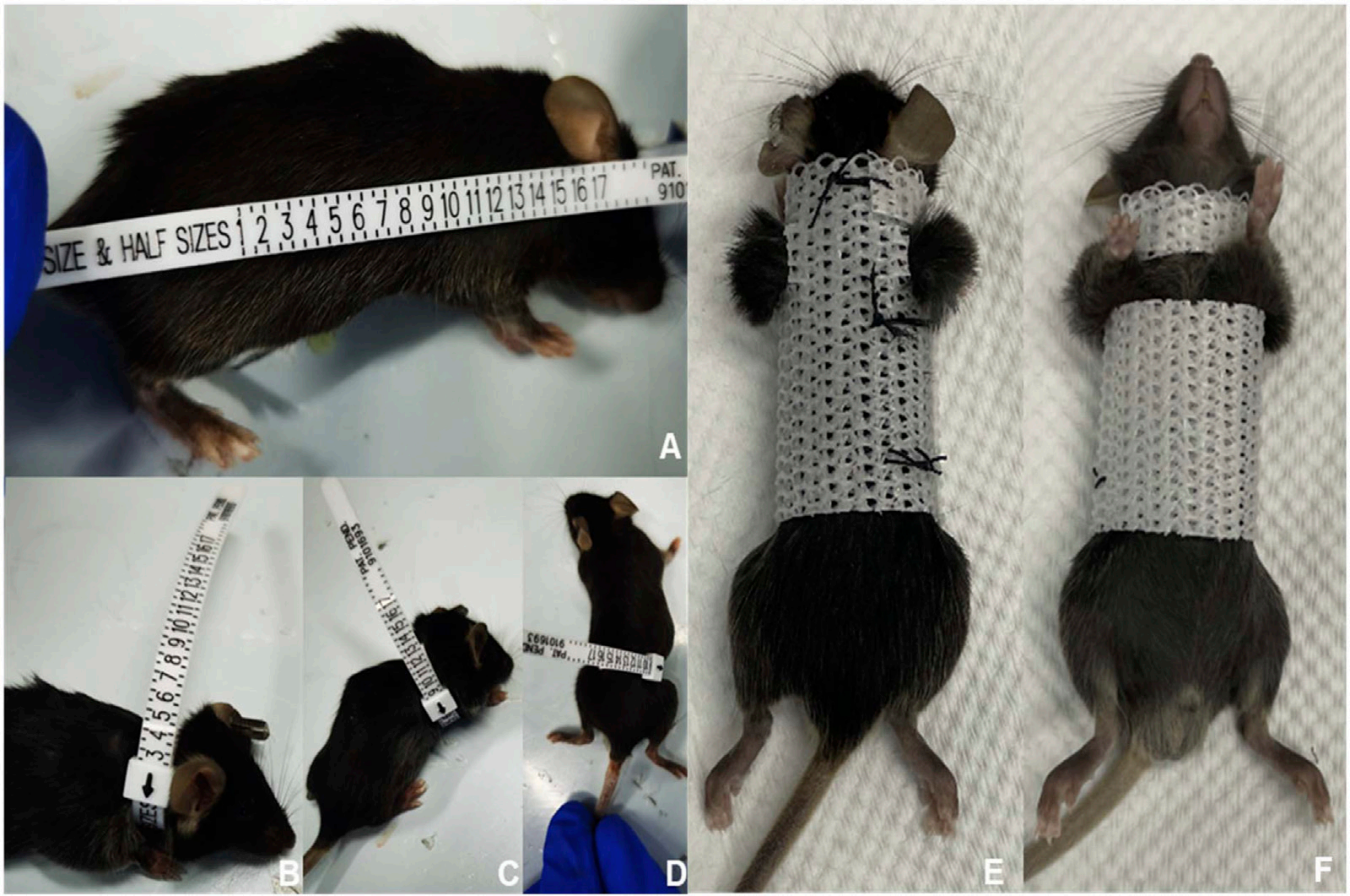
Process of constructing and using the external fixed support. First, we measured and recorded the size of various body parts of the mice **(A–D)**. Next, we designed and fabricated an external fixation device that was tailored to fit the mouse’s body size **(E,F)**. When the mouse wore the external fixation device, its upper limbs and shoulder joints were left exposed to avoid interfering with drinking and eating.

A polyester fiber material was employed to fabricate external fixation braces for the mice. Prior to fitting the braces, measurements of body length, neck, chest, and abdominal circumference of the mice were taken, which were then used to custom-cut the fixation brace. The mice were placed under general anesthesia during the fitting process, and both forelimbs (including the shoulders) were exposed outside the brace to enable unrestricted movement (Figure 2; Supplementary Videos S1, S2). To ensure optimal physical development, the fixation device was replaced once a week. To evaluate the brace’s impact on spinal flexion and extension, mice were placed in plastic bottles and subjected to X-ray examination to determine the degree of spinal flexion and calculate the sagittal Cobb angle.

### 2.4 Treadmill training program

**FIGURE 3:**
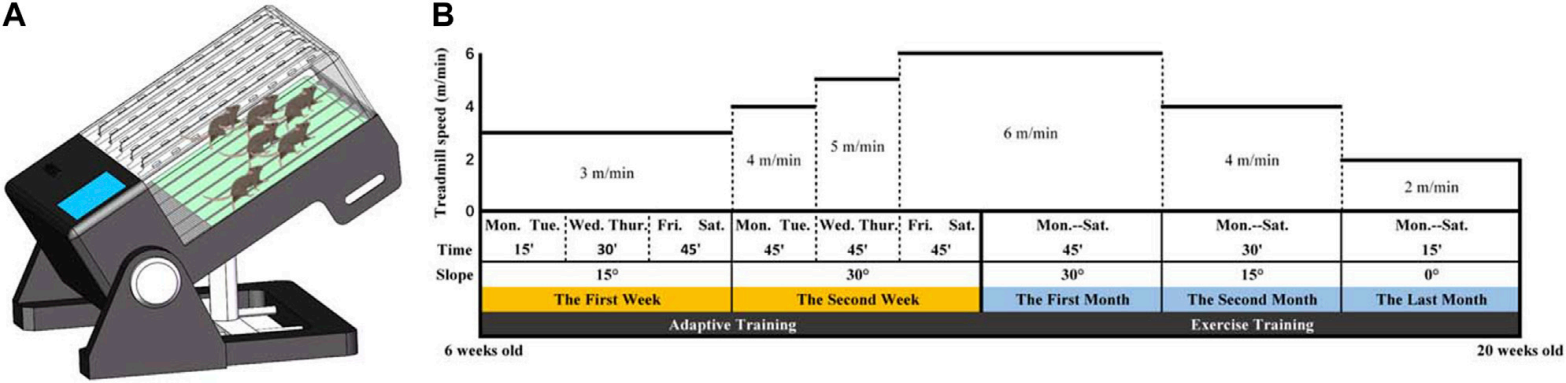
**(A)** A treadmill with an adjustable runway slope was used for mouse running training. This treadmill allowed six mice to undergo running training simultaneously. **(B)** Running training programs.

A treadmill equipped with track-slope adjustment capabilities was utilized for the running training of mice (Figure 3A; Supplementary Material S2). This apparatus enabled six mice to undergo running training simultaneously, with each track being independent so that every mouse could achieve independent running speeds. Gene-deficient mice commenced treadmill training at 6 weeks old and were trained 6 days per week (Monday to Saturday). A suitable running training plan, adjusted according to the exercise intensity that mice of different ages could handle and based on previous literature (Wu et al., 2020), was implemented. All mice underwent adaptive training in the first 2 weeks, during which the running speed gradually increased from 3 to 6 m/min, the slope angle gradually increased from 0° to 30°, and the training duration gradually increased from 15 to 45 min. Subsequently, the mice underwent regular training for 3 months, during which the training time gradually decreased from 45 to 15 min, the speed gradually decreased from 6 to 2 m/min, and the slope gradually decreased from 30° to 0°. However, as the training program continued, a combination of an aggravated secondary SCI and calcification of the Achilles tendon led to severe gait changes and the eventual inability to run. Our previous observations indicated that mice were unable to tolerate longer running training beyond 20 weeks old. Given that finding, we set 20 weeks old as the endpoint of the training program (Figure 3B).

### 2.5 Micro-CT image acquisition and analysis

To investigate the process of HOTL, mice were assessed at different ages using micro-CT analyses (SCANCO MEDICAL, μCT100, Bassersdorf, Zurich, Switzerland). The data were analyzed at a threshold of 255 for the detection of ectopically mineralized components. Reconstructed images were analyzed by CTvol (version 3.2.0, Bruker Corp., United States), Data Viewer (version 1.5.3.4, Bruker Corp., United States), and Xiaosai Viewer (version 3.2.1, China) software.

### 2.6 Tissue specimen acquisition and processing

At designated time points, mice were sacrificed by inhaling an overdose of isoflurane. Subsequently, they were perfused with phosphate buffered saline (PBS), followed by 4% paraformaldehyde (PFA). The entire spine and bilateral lower limbs were dissected and immersed in 4% PFA overnight. After being washed with PBS, bone tissue was decalcified using 12.5% ethylene diamine tetraacetic acid disodium (DD0002, Leagene, China) for 5–7 days. The decalcified tissue was then washed with PBS and dehydrated by immersion in 20% sucrose overnight. Finally, the tissues were embedded in optimal cutting temperature compound. (Sakura, Japan) and stored at −20°C. To visualize tissues clearly, representative frozen sections (10 μm thick) were obtained from the sagittal and axis views of the spine, as well as the sagittal view of the lower limbs.

### 2.7 Hematoxylin and eosin (HE) staining

A commercial hematoxylin and eosin staining kit (Beyotime, C0105S, China) was used according to the manufacturer’s instructions. Briefly, after rehydration, the frozen sections were fixed with 4% PFA for 15 min, followed by immersion in hematoxylin for 10 min and eosin for 1 min. Subsequently, the sections were dehydrated in varying concentrations of ethanol. The results were visualized using a digital pathology section scanner (KF-PRO-005, KFBIO Technology).

### 2.8 Immunofluorescence analysis

After being brought to room temperature and rehydrated with PBS for 15 min, frozen sections were permeabilized with 0.3% Triton X-100 (Sigma, America) for 30 min and blocked with 5% bovine serum albumin (BioFroxx, Germany) for 1 h. Subsequently, the sections were incubated overnight at 4°C with a YAP antibody (Abcam, 1:1000, Britain) diluted in PBS. After washing with tris buffered saline tween three times for 5 min each time, the tissue sections were incubated with goat anti-rabbit IgG in the dark for 1 h. Finally, nuclei were marked with 6-diamidino-2-phenylindole (DAPI, Abcam, Britain). The fluorescence images were acquired using confocal microscopy (Carl Zeiss, Germany).

### 2.9 Behavioral evaluation of Basso-Beattie-Bresnahan (BBB) scores

Hindlimb motor function was assessed at various observation points using BBB locomotion scale. The BBB locomotion scale ranges from 0 (complete hind limb paralysis) to 21 (normal locomotion) points according to walking ability, hindlimb joint movements, and coordination. The evaluation was conducted by two blinded observers who were unaware of the experimental conditions.

### 2.10 Statistical analysis

Statistical analysis was performed using SPSS version 20.0 (SPSS, United States). All values are reported as means ± standard deviation. The normality of the data distribution was evaluated using the Shapiro‒Wilk normality test. Student’s t-test (for two groups) or one-way ANOVA (for more than two groups) was used to determine statistical significance (*p* < 0.05). In cases of nonnormally distributed data, the Mann‒Whitney *U* test was employed.

## 3 Results

### 3.1 Observation of the beginning time and site of OPLL in *EIIa* ^*cre*^ *-Enpp1* ^*flox/flox*^ mice

**FIGURE 4:**
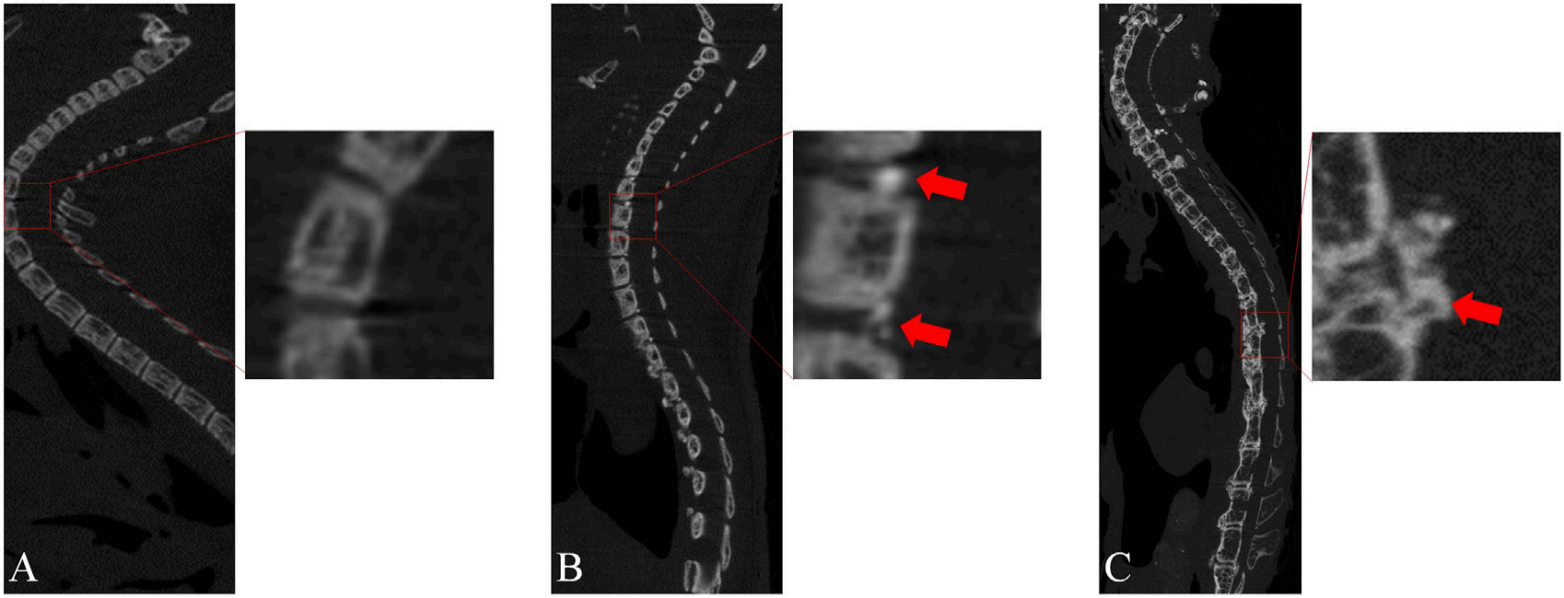
Micro-CT sagittal images of genetically defective mice at different ages. **(A)** At 4 weeks of age, almost no OPLL was observed. **(B)** At 8 weeks of age, a minor degree of heterotopic ossification was observed in the PLL located behind the intervertebral space. **(C)** At 28 weeks of age, a significant degree of heterotopic ossification was noted behind the intervertebral space, accompanied by a minor amount of heterotopic ossification behind the vertebral body.

To identify the onset of OPLL in mice and enable early intervention, we conducted continuous micro-CT examinations on mice between 4 and 28 weeks of age (Figure 4). Our findings showed that at 4 weeks of age, there was hardly any ossification observed in the spine of mice, and only a few ossification lesions were detected behind the vertebral space at 8 weeks of age. Therefore, we decided to initiate the intervention at 6 weeks of age. From the micro-CT results, we observed that pathological changes in OPLL behind the vertebral space were significantly greater than those behind the vertebral body from 8 to 28 weeks of age. We speculate that mechanical stimulation may play a significant role in this process, given that the PLL behind the intervertebral space exhibits considerably more motor activity than that behind the vertebral body.

### 3.2 YAP exhibits high expression levels in cases of heterotopic ossification affecting the spinal ligament and Achilles tendon

**FIGURE 5:**
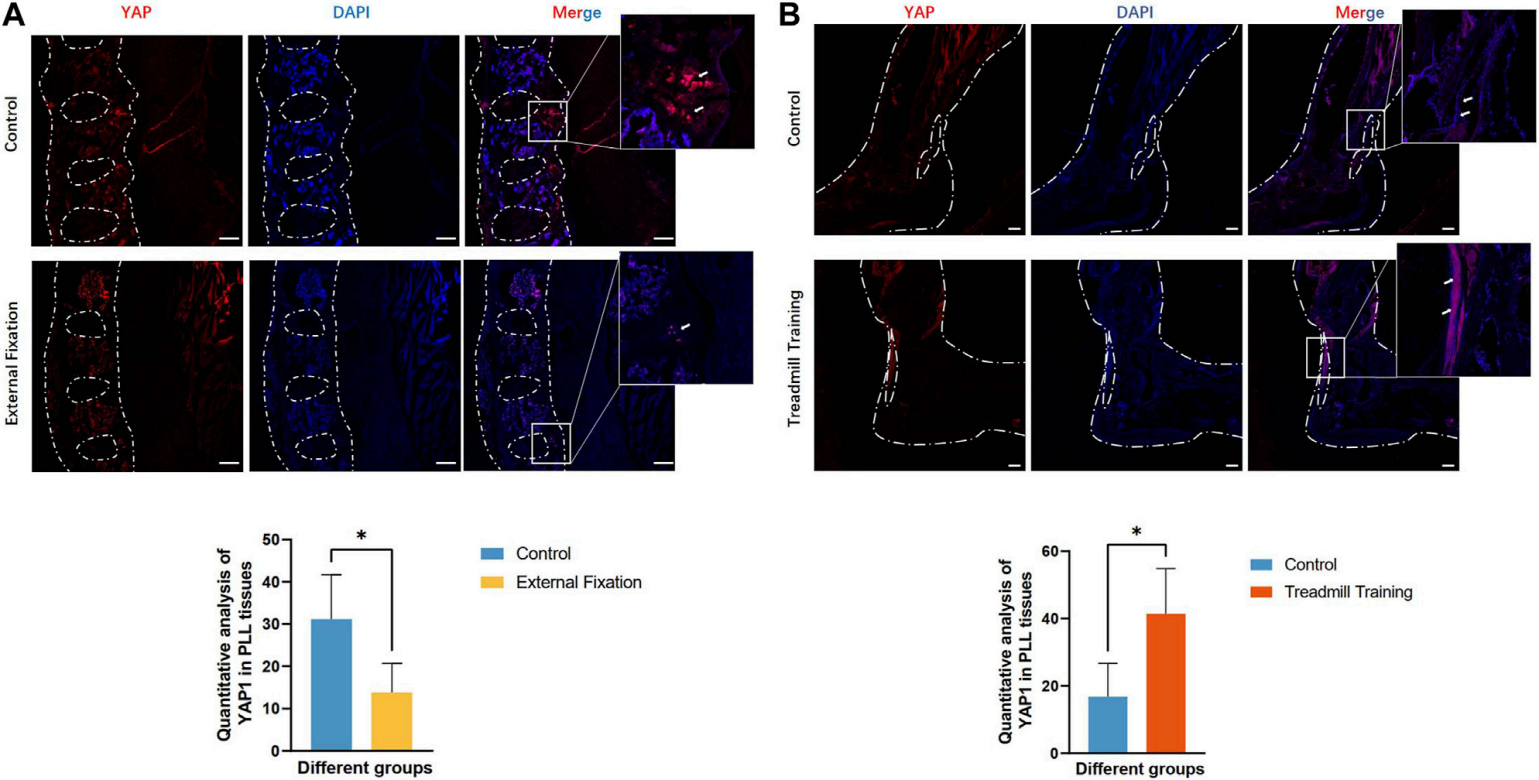
Expression of YAP protein in the PLL and Achilles tendon. **(A)** The expression of YAP was lower than that of the control group. **(B)** The expression of YAP was greater than that of the control group.

Immunofluorescence staining was employed to detect the expression of YAP in tissues. In the sagittal position of the spine, normal PLL tissues displayed lower levels of YAP expression, whereas the ossification site exhibited higher expression levels. Consistent with the micro-CT findings, the heterotopic ossification region in the PLL of *EIIa* ^*cre*^ *-Enpp* ^*flox/flox*^ mice without any treatment was larger than that in the external fixation group. Accordingly, higher levels of YAP expression were detected in the region with greater heterotopic ossification (Figure 5A). Similar results were observed in the Achilles tendon, with significantly lower YAP expression in the control group than in mice subjected to treadmill training (Figure 5B). As a mechanotransduction response factor, increased YAP expression implies greater mechanical stimulation in the affected area.

### 3.3 Measurement of the spinal Cobb angle in mice while in a standing position

**FIGURE 6:**
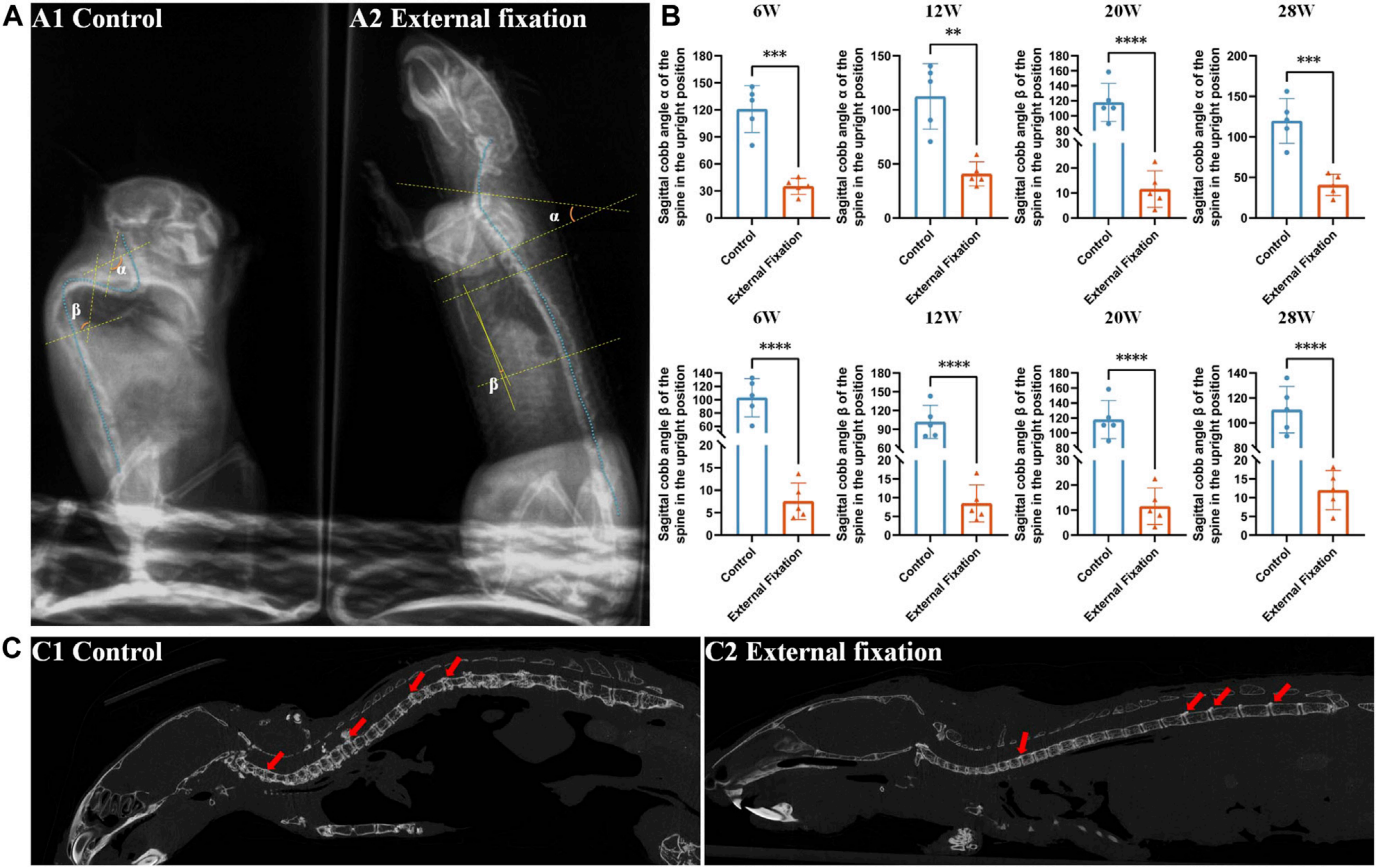
Effect of wearing an external fixation brace on the progression of heterotopic ossification. **(A)** The sagittal Cobb angle in mice was measured by X-ray examination. A1: control group; A2: external fixation group. **(B)** Significant differences were observed between the external fixation group and the control group at all observation time points. **(C)** The micro-CT sagittal images of the mice at 28 weeks of age revealed that the control group maintained the physiological curvature of the spine, while the external fixation group exhibited almost straight alignment.

The sagittal Cobb angle was utilized to evaluate the flexion and extension range while the mice were standing. As illustrated in Figure 6A, there was a significant difference in the degree of spinal Cobb angle between groups in the cervicothoracic and lower thoracic segments when the mice were upright. For clarity, we designated the Cobb angle in the cervicothoracic segment (C5-T4) as the *α* angle and the Cobb angle in the lower thoracic segment (T6-T12) as the *ß* angle.

Both the *α* angle and *ß* angle of the control group were greater than those of the external fixation group, with a statistically significant difference (*p* < 0.01). The Cobb angle of mice without an external spinal brace was almost obtuse, with a mean value greater than 90° at each time point. On the other hand, the angle of mice in the fixed group was almost straight, with a mean angle less than 30° (*p* < 0.01, Figure 6B).

The micro-CT findings indicated that even when the mice reached 28 weeks of age, the external spinal brace still imposed restrictions on spinal rotation. At this stage, the spine was almost straight, except for the minor physiological curvature in the cervicothoracic segment. A considerable amount of heterotopic ossification was observed behind the nonimmobilized intervertebral space in the thoracolumbar region and to a lesser extent behind the cervical and thoracic vertebrae (Figure 6C).

According to this result, our subsequent experimental observations focused on and compared heterotopic ossification in the cervicothoracic segment (C5-T4) and the lower thoracic segment (T6-T12).

### 3.4 Micro-CT was utilized to quantitatively compare the size of the OPLL among different groups

Sagittal radiographs of the spine revealed substantial differences in Cobb angles between the external fixation model and the control group in the cervicothoracic segment (C5-T4) and lower thoracic vertebrae (T6-T12). Thus, micro-CT was utilized to quantitatively analyze the amount of ligament ossification and compare the size of the OPLL behind the intervertebral disc spaces. Since mice could not endure extended running training beyond 20 weeks of age, the treadmill training group was only evaluated at this time point.

**FIGURE 7:**
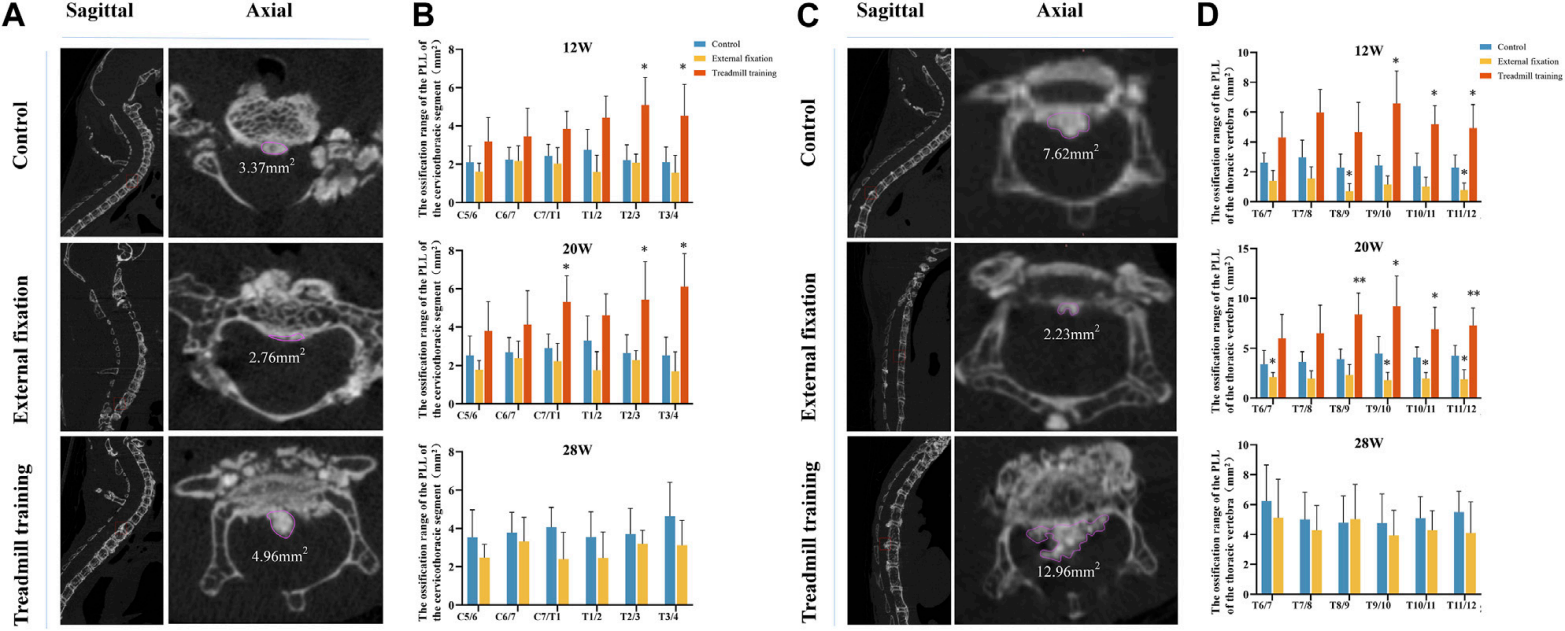
Micro-CT was utilized to compare the extent of OPLL. **(A,B)** Comparison of the size of ossification in the cervicothoracic segment (C5-T4) of the intervertebral space among different groups of mice at varying ages. **(C,D)** Comparison of the size of ossification in the cervicothoracic segment (C5-T4) of the intervertebral space among different groups of mice at different ages.

No significant difference was observed in the range of OPLL between the external fixation group and the control group from 12 weeks to 28 weeks (*p* > 0.05). However, at 12 weeks, the ossification range of T2/3 and T3/4 in the treadmill training group was significantly greater than that in the control group. At 20 weeks of age, the ranges of C7/T1, T2/3, and T3/4 in the treadmill training group were significantly greater than those in the control group (*p* < 0.05, Figures 7A, B).

Comparison of the lower thoracic segment between the groups demonstrated that at 12 weeks of age, the ossification range of the T8/9 and T11/12 intervertebral spaces in the external fixation group was significantly smaller than that in the control group, while the range of the T9-T12 intervertebral space in the running group was larger than that in the control group (*p* < 0.05). At 20 weeks of age, the ossification range of the T6/7 and T9-T12 intervertebral spaces in the external fixation group was significantly smaller than that in the control group, while the range of T8-T12 in the treadmill training group was larger than that in the control group (*p* < 0.05). At 28 weeks of age, there were no significant differences in the ossification range between the external fixation group and the control group (*p* < 0.05, as shown in Figures 7C, D).

### 3.5 Effects of treadmill training on gait in mice

Micro-CT analysis confirmed that exercise accelerates the progression of OPLL, which could potentially worsen secondary spinal cord injury.

**FIGURE 8:**
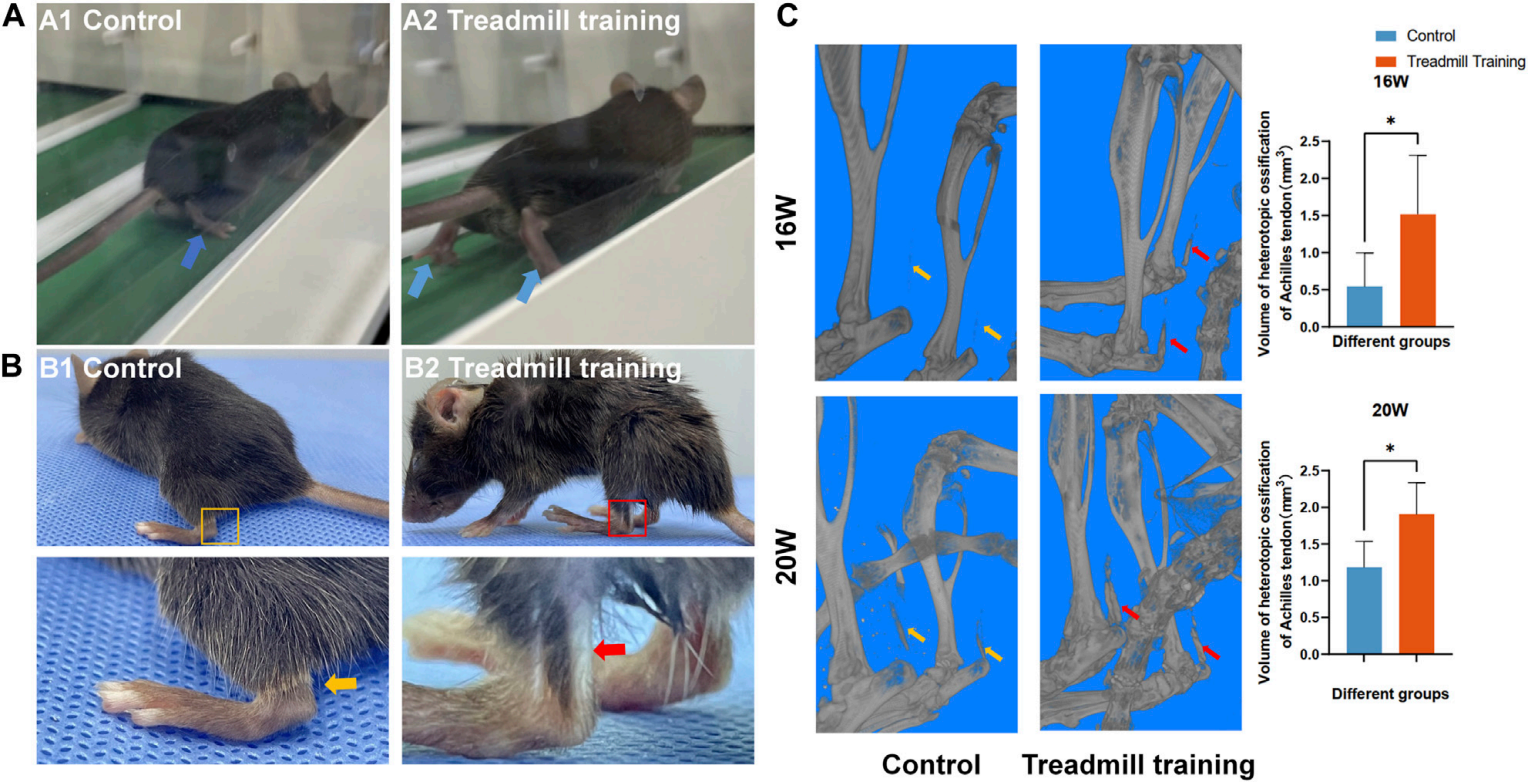
Effect of treadmill training on the gait of mice. **(A)** Gait differences of mice in different groups while running. A1: Control group mice landed on the soles of their feet. A2: Treadmill training group mice landed on their tiptoes. **(B)** At 20 weeks of age, white strip-like changes were observed in the Achilles tendon of the hind limbs in the treadmill training group. **(C)** The micro-CT 3D reconstruction revealed that the extent of ossification in the Achilles tendon of the treadmill training group was significantly greater than that of the control group at 16 and 20 weeks of age.

As shown in Figure 8A, with the dual pathological changes of chronic spinal cord compression and Achilles tendon ossification, the mice gradually switched from using the sole to using the tip toe when walking. As these two negative effects grew worse, the walking ability of the mice was weakened. By the time the mice were 20 weeks old, the ankle joint of their hind limb had become almost rigid and fixed at a 90° angle. To maintain their balance, the mice could only land on their heels. Additionally, there was a white strip in the Achilles tendon of the hind limb, indicating serious ossification changes in that area (Figure 8B). Micro-CT 3D reconstruction images showed that the degree of Achilles tendon ossification in the control group was significantly lower than that in the treadmill training group at 16 and 20 weeks of age (Figure 8C, *p* < 0.05).

### 3.6 HE staining was utilized to observe the extent of ossification in the PLL

**FIGURE 9:**
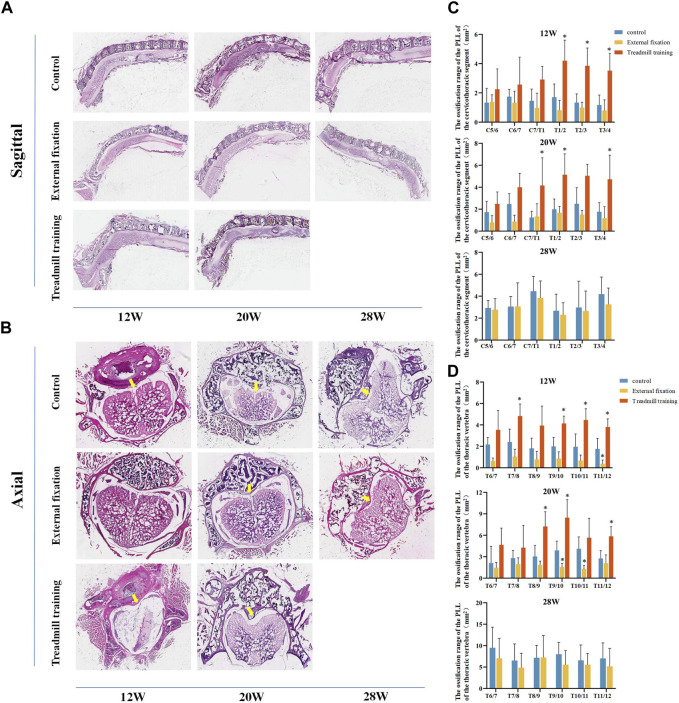
The extent of ossification in the PLL was assessed using HE staining. **(A,B)** HE staining in different groups. **(C)** Quantitative analysis was performed to compare the range of ossification in the cervicothoracic segment among the three groups of mice. **(D)** Quantitative analysis was performed to assess the range of ossification in the lower thoracic section of mice in the three groups using HE staining.

HE staining was employed to compare the size of OPLL between different groups. Similar to micro-CT, we aimed to compare the extent of ossification in the cervicothoracic segment (C5-T4) and the lower thoracic segment (T6-T12) (Figures 9A, B).

From 12 weeks to 28 weeks of age, there were no noteworthy differences in the extent of ossification in the PLL in the cervicothoracic segment between the external fixation group and the control group (*p* > 0.05). However, the range of T1-T4 in the treadmill training group was notably greater than that of the control group at 12 weeks old. At 20 weeks of age, the ossification range of C7/T1, T1/2, and T3/4 in the treadmill group was significantly greater than that of the control group (*p* < 0.05, Figure 9C).

The HE staining results in the lower thoracic segment indicated that at 12 weeks of age, the ossification range of the T11/12 ligament was significantly smaller in the external fixation group than in the control group, while the ossification range of T7/8 and T9-T12 was larger in the treadmill training group than in the control group (*p* < 0.05). At 20 weeks of age, the ossification range of T9-T11 was significantly smaller in the external fixation group than in the control group, while the range of T8-T10 and T11/12 was larger in the treadmill group than in the control group (*p* < 0.05). At 28 weeks of age, there was no significant difference between the external fixation group and the control group (*p* > 0.05, Figure 9D).

In summary, the findings from HE staining were consistent with those obtained from micro-CT.

### 3.7 Ratio of the extent of calcification in the Achilles tendon

**FIGURE 10:**
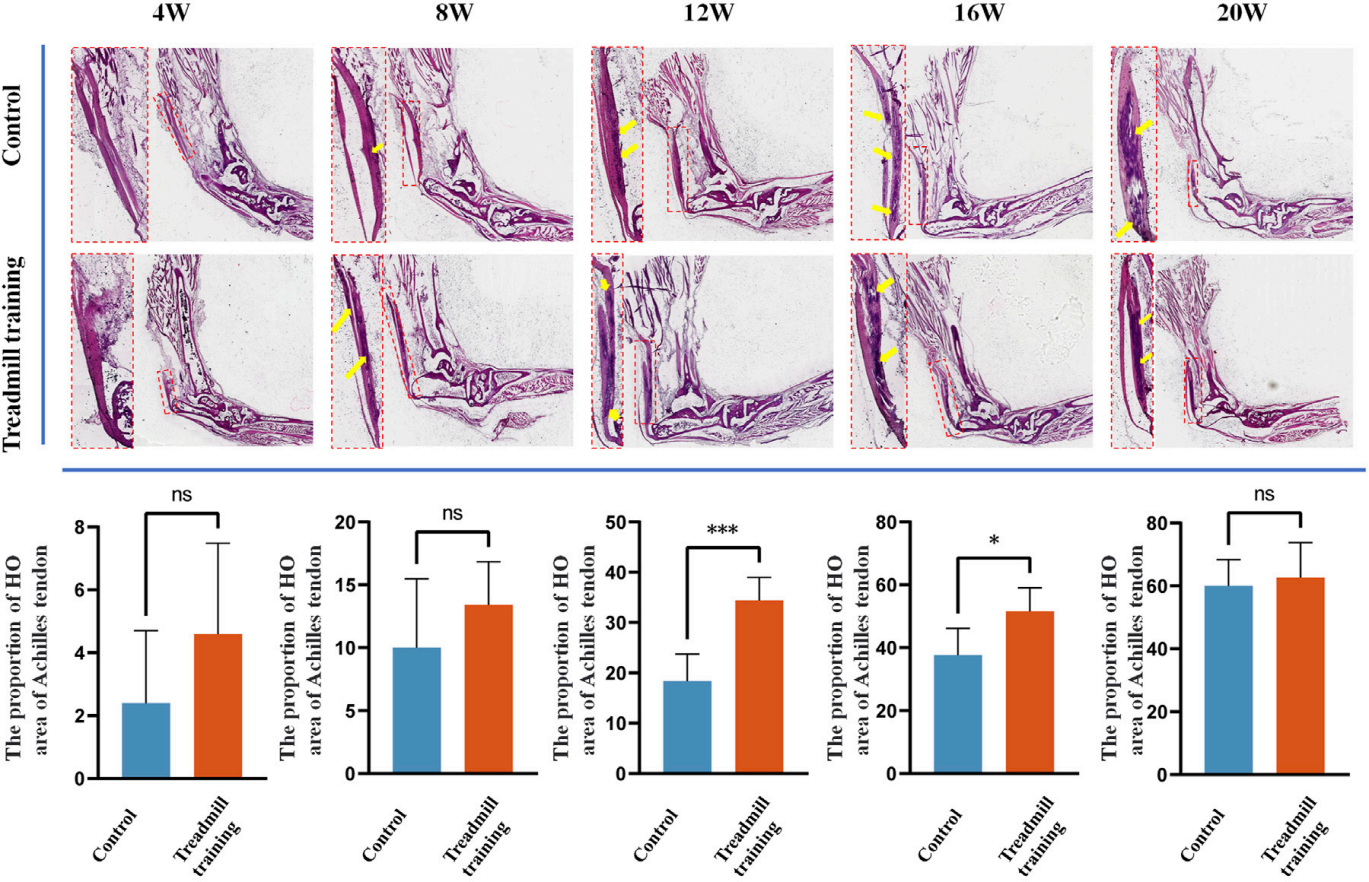
HE staining was performed on the Achilles tendon in the running training group and the control group. The results showed that at 12 weeks and 16 weeks of age, the range of Achilles tendon ossification was significantly larger in mice undergoing running training than in the control group. At 20 weeks of age, both groups exhibited significantly expanded ossification areas in the Achilles tendon, but there was no statistical significance between them.

We also investigated the effects of treadmill training on heterotopic ossification of the Achilles tendon in mice, focusing on the proportion of the ossification area in the whole tendon (Figure 10). Both groups of mice exhibited ossification of the Achilles tendon from 8 weeks of age. At 12 and 16 weeks of age, the area of Achilles tendon ossification in the treadmill training group was significantly larger than that in the control group (*p* < 0.05). However, at 20 weeks of age, there was no significant difference in the ossification area between the two groups (*p* > 0.05).

### 3.8 Effects of various treatments on chronic spinal cord injury in mice

**FIGURE 11:**
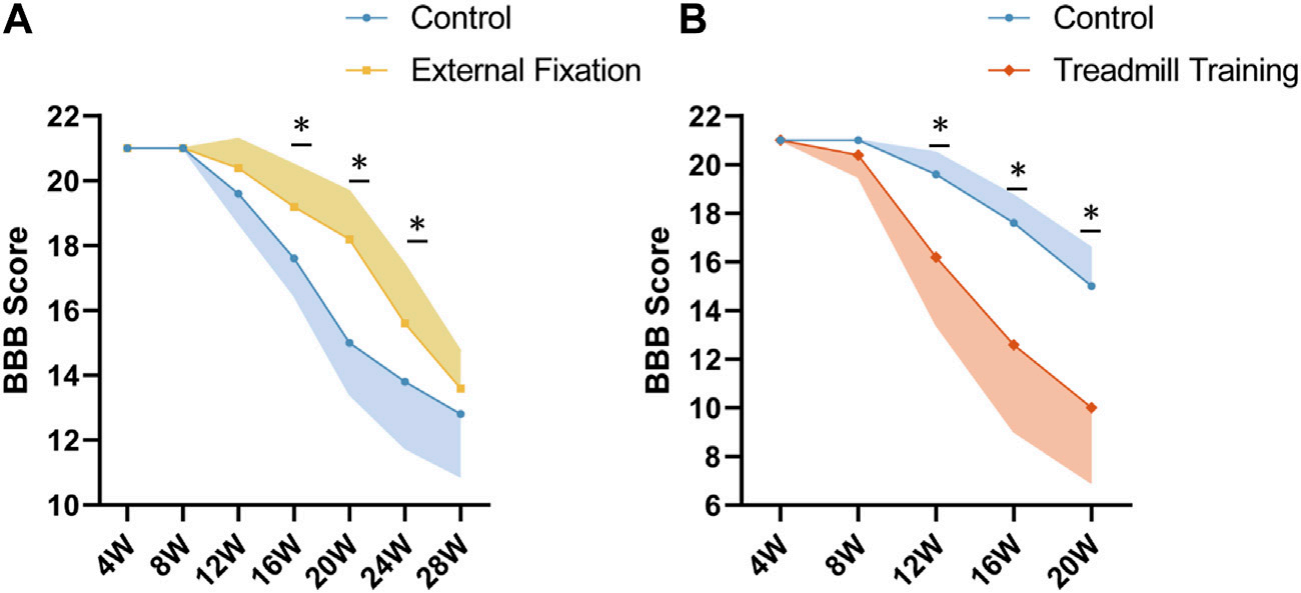
BBB scores among different groups. **(A)** At 16 and 20 weeks of age, the BBB scores of spinal immobilized mice were significantly higher than those of the control group, indicating that spinal immobilization may have a positive effect on recovery from spinal cord injury. **(B)** From 12 to 20 weeks of age, the BBB score of mice undergoing treadmill training was significantly lower than that of the control group, suggesting that treadmill training may have a negative impact on recovery from SCI.

The BBB score was used to assess the severity of chronic SCI in genetically deficient mice. External fixation was found to alleviate chronic SCI, as evidenced by significantly higher BBB scores in the external fixation group than in the control group at 16, 20, and 24 weeks of age (*p* < 0.05, Figure 11A). However, there was no significant difference between the two groups at 28 weeks of age. The study also revealed that treadmill training accelerated the ossification process, leading to a faster progression of chronic spinal cord injury. Mice undergoing treadmill training had a significantly lower BBB score than the control group after 12 weeks of age (*p* < 0.05, Figure 11B).

## 4 Discussion

Experimental animals are biological models obtained through carefully designed experiments that offer a high degree of uniformity. They allow researchers to study the pathophysiological mechanisms of diseases and potential treatments more accurately. Previous research has shown that mechanical stimulation has a significant impact on cell osteoblastic differentiation at the cellular level, and this process is associated with the nuclear translocation of the mechanical signal response factor YAP (Zhu et al., 2023). In this study, we aimed to establish an appropriate animal model that can serve as a basis for further *in vivo* investigation of this mechanism.

To our knowledge, few animal models that address the mechanical factors that promote the progression of heterotopic ossification have been reported. Tsukamoto’s team designed a device that could apply circular stretching to the rat’s caudal ligament (Tsukamoto et al., 2006). The rat’s pelvis was secured to a distal fixator with a steel pin inserted into the 8th caudal vertebra, while its body was restrained on the edge of a moving bed that moved back and forth to exert twice its body weight on the anterior tail segment (2-5 caudal vertebrae). Histological staining revealed that when this traction was sustained for 2 weeks, heterotopic ossification occurred near the intervertebral space in the stressed area of the coccygeal ligament. A significant number of mature chondrocyte-like cells appeared near the ossification area, and the duration of stretching time had a positive correlation with these pathological changes.

Zhao et al. (2021) developed a device capable of applying stretch stress to the ligamentum flavum in the thoracolumbar segment of rats. They immobilized the rats’ limbs in parallel splints, with their chests and abdomens pressed against elastic boards. When the device was turned on, an eccentric wheel drove the elastic board to periodically flex and extend the thoracolumbar vertebrae, thereby forcing the ligamentum flavum to bear cyclic tensile stress. Four weeks of cyclic stimulation induced heterotopic ossification of the ligamentum flavum at the stress site of the thoracolumbar region. Further *in vitro* verification indicated that this process might be associated with the high expression of integrin β3 induced by stretch stimulation.

Histological staining and micro-CT imaging results from previous animal models have confirmed the ability of mechanical factors to induce heterotopic tissue ossification in animals. However, non-traumatic heterotopic ossification in clinical cases often involves multiple pathogenic factors, making it difficult to simulate the complete pathological process of the disease with a single mechanical stimulus factor. Therefore, there is a need to establish more reasonable animal models. Previous clinical sequencing results have shown that the expression of the *ENPP1* gene in the peripheral blood of OPLL patients was significantly lower than that of normal individuals (Saito et al., 2011; Mehta et al., 2012; Ferreira et al., 2021; Kato et al., 2022), and naturally mutated tiptoe walking (ttw) mice have been shown to have mutations in the gene site of *Enpp1* (Mackenzie et al., 2012). Building on this, our research group constructed *EIIa* ^*cre*^ *-Enpp1* ^*flox/flox*^ gene-defective mice using CRISPR‒Cas9 technology. These mice exhibited clinical symptoms similar to those of OPLL patients, including spontaneous ectopic bone formation, gradual spinal cord compression, and the onset of chronic SCI symptoms such as unsteady gait and difficulty walking. With this gene-defective mouse model, we can study the mechanism of mechanical stimulation in the progression of OPLL and propose corresponding intervention measures according to these mechanical factors.

Micro-CT imaging revealed that OPLL originated behind the intervertebral space and appeared relatively late behind the vertebral body. This difference in position may be due to variations in the stress placed on the ligaments. The PLL located in the intervertebral space is subjected to tensile stress due to the spine’s flexion and extension, while the PLL close to the vertebral body is subjected to less tensile stress due to its close connection to the vertebral body. Furthermore, immunofluorescence staining indicated a significant increase in YAP expression at the site of ligamentous heterotopic ossification, which is consistent with previous *in vitro* studies (Zhu et al., 2023). This finding suggests that mechanical stimulation plays a vital role in the pathological process.

Some scholars have also explored targeting mechanical factors as a new approach to delaying ossification. Previous studies have mainly focused on therapeutic measures for the gene mutation itself. For example, Hiratsuka et al. supplemented levamisole and exogenous inorganic pyrophosphate (PPi) in ttw mice to maintain a high level of serum PPi, thus delaying the progression of heterotopic ossification (Hiratsuka et al., 2018). Additionally, some studies have shown that supplementing ttw mice with exogenous *Enpp1* recombinant enzyme (INZ-701) can maintain blood PPi at physiological levels for 3 consecutive days and that continuous medication for 8 weeks can effectively prevent heterotopic ossification. Furthermore, H2 receptor antagonists (Maeda et al., 2015), nuclear retinoic acid receptor *γ* agonists (Hiratsuka et al., 2018) and neurotrophic factors (Uchida et al., 2012) have also been reported to delay heterotopic ossification or alleviate secondary chronic SCI in ttw mice. Although these therapeutic measures may have clinical efficacy, the ethics, safety, and side effects associated with drug use make them difficult to use in the clinic in the short term. Therefore, safer and more feasible therapeutic approaches need to be considered.

To delay the progression of heterotopic ossification of the spinal ligaments, we wanted to limit the movement of the spine. To achieve this, we designed an external spinal fixation brace, which is the first of its kind to be used in mice. The brace limits spinal flexion and extension, thereby reducing ligament stretching stress. The weight of the external fixation brace was only 1–1.5 g, which is approximately 1/20 of the body weight of mice, and had no adverse effect on physiological activities. Additionally, the external fixation brace was replaced weekly to accommodate the changing body size of the mice. When wearing the brace, the double forelimbs were left fully exposed to avoid interfering with eating and drinking.

We observed that during normal physiological activities in mice, spinal flexion and extension were used to maintain body balance, especially during standing and running. X-ray images showed that when mice were in a standing position, the cervicothoracic segments and the lower thoracic segments were in a state of hyperflexion. However, when mice wore the external fixation brace, their spinal mobility was significantly limited. While this animal model did not show a significant effect in delaying ossification in the cervicothoracic segment, it did show such an effect in the lower thoracic segment. There may be several reasons for this phenomenon. First, the external fixation brace was made by cutting and curling flat material, and several layers were wrapped around the abdomen of the mice to increase its mechanical strength, while there was only one layer around the cervical spine. Second, in addition to flexion and extension, the neck can also rotate, but the external fixation brace could not effectively limit the rotation of the neck. Conversely, the restrictions on the limbs made it difficult for the mice to make any abdominal movements.

To further evaluate the impact of changes in the ossification range on pathological signs in mice, we utilized the BBB score for SCI to observe the mobility of the hind limbs of mice. The results indicated that the external fixation group was able to effectively protect against SCI from 16 to 24 weeks of age, as evidenced by a significantly higher BBB score than that of the control group. However, by 28 weeks of age, there was no significant difference in BBB scores between the two groups. We speculated that the deletion of the *Enpp1* gene might be a contributing factor to the mice’s strong ability to spontaneously develop ligamentous heterotopic ossification. While limiting spinal movement could delay the process of heterotopic ossification, it had no effect on the treatment or reversal of ossification.

In this part of the experiment, we also aimed to evaluate the impact of physical hyperactivity, specifically running, on the progression of ossification. The effect of physical activity on patients with heterotopic ossification remains a controversial topic in the medical community. While some scholars have suggested that passive limb exercise can effectively delay traumatic heterotopic ossification (Vasileiadis et al., 2021; Wang et al., 2021; Vasileiadis et al., 2023), others have concluded that repeated stress stimulation of the spine can accelerate ossification progression and negatively impact patients’ quality of life (Lee et al., 2017; Kang et al., 2019; Lee et al., 2021). Additionally, the benefits of appropriate physical exercise for the recovery of neurological function in OPLL patients have not been uniformly established. Some experts recommended running exercise as an essential rehabilitation measure for the recovery of spinal cord function after chronic SCI, while others argued that mechanical stimulation from running may not only increase the size of the ossification range but also cause additional damage to the spinal cord tissue (Wang et al., 2015; Yu et al., 2019; Shibata et al., 2021). Repeated flexion and extension of the spine may lead to a smaller spinal canal volume, increasing the compression of the spinal cord (Azuma et al., 2010; Ito et al., 2015). Moreover, the uneven surface of the protruding site of ossified tissue can cause direct physical damage to the spinal cord, leading to secondary inflammatory reactions (Fournely et al., 2020; Jannesar et al., 2021).

This study aimed to investigate whether excessive physical activity could accelerate the progression of ossification by introducing treadmill training. The intensity of the training was gradually increased by adjusting the speed, slope, and running time. Micro-CT results revealed a significant expansion of ossification in specific segments of the mice undergoing treadmill training compared to the control group. Additionally, the BBB score of the treadmill group at 12 weeks of age was significantly lower than that of the control group. These findings are consistent with previous reports indicating that repeated flexion and extension of the spinal canal may aggravate spinal cord compression when ossification compression is not relieved (Lee et al., 2017; Kang et al., 2019; Lee et al., 2021). Further experimental verification is needed to determine whether the aggravation of SCI is due to direct mechanical injury or secondary inflammatory reactions.

This study has several limitations that need to be acknowledged. First, while radiographs showed that the external fixation brace effectively limited spinal movement and micro-CT demonstrated delayed progression of lower thoracic OPLL, the brace was found to be less effective than internal fixation due to soft tissue cushioning. Second, the accuracy of HE staining of axial sections was limited by frozen section technology, making it difficult to position the staining precisely behind the intervertebral space. Therefore, the results of HE staining can only be used to supplement the findings of micro-CT. Third, owing to the diminutive size of mice, our current approach was confined to employing external fixation solely for the purpose of restricting spinal movement (from cervical to lumbar regions) rather than applying any alternative interventions aimed at curbing activity in other body areas, such as the limbs. Finally, the genetically defective mice used in this study develop ossification of ligament tissue throughout the whole body, rather than just in the PLL or Achilles tendon, which may affect experimental observations to some extent.

## 5 Conclusion

In this study, we designed an external fixation device to limit the flexion and extension of the spine and reduce the ossification progress of PLL in mice with *Enpp1* gene deficiency. Additionally, we used treadmill training to promote heterotopic ossification of the PLL and Achilles tendon. Our *in vivo* experiment confirmed that mechanical stimulation is an important factor in the progression of OPLL. These rational animal models provided an experimental basis for further study of the specific pathological mechanism. Furthermore, we confirmed that YAP, a transcriptional coactivator protein, plays a crucial role in accelerating the process of heterotopic ossification of ligaments by mechanical stimulation. However, more *in vivo* studies are necessary to confirm these results.

## Data Availability

The original contributions presented in the study are included in the article/Supplementary Materials, further inquiries can be directed to the corresponding authors.

## Supplementary material

SUPPLEMENTARY FIGURE S1 Genotypic identification of mice by agarose gel electrophoresis.

SUPPLEMENTARY FIGURE S2 Animal treadmill.

SUPPLEMENTARY VIDEO S1 Wearing an external fixation brace did not have any significant effect on the dietary intake of the mice.

SUPPLEMENTARY VIDEO S2 Wearing an external fixation brace did not have any significant impact on the daily activities of the mice.

## Abbreviations

BBB, Basso-Beattie-Bresnahan; DAPI, 6-diamidino-2-phenylindole; HE, hematoxylin and eosin; HOTL, heterotopic ossification of tendons and ligaments; OPLL, ossification of the posterior longitudinal ligament; PBS, phosphate buffer solution; PFA, paraformaldehyde; PLL, posterior longitudinal ligament; PPi, inorganic pyrophosphate; SCI, spinal cord injury; ttw, tiptoe walking; YAP, yes-associated protein.
