# Supplementary material for: Effect of mechanical stimulation on tissue heterotopic ossification: an in vivo experimental study
Source: Front Physiol. 2023 Oct 11;14:1225898. doi: 10.3389/fphys.2023.1225898 (PMC10600381; doi:10.3389/fphys.2023.1225898)
Supplement: Supplementary file 1 [file Table1.DOCX]

**Genotype identification of mice**

Standard genotyping protocols were used to genotype the mice, which included sequencing analysis. At 2 weeks old, tail fragments of 2 mm were clipped, digested with the MG500 mouse genotyping kit (Genecopoeia), and then subjected to DNA amplification. PCR was conducted on a Real-Time System (Bio-Rad) using Pro Taq Master Mix dye plus (AG11112, ACCURATE BIOLOGY AG, China). Each reaction mixture contained 1 μl of the cleaved product, 12.5 μl of 2 × Master Mix, 10.5 μl of nuclease-free water, and 0.5 μl each of 10 μM. The specific primers used in this study were designed using Primer 6.0 software (Applied Biosystems, Foster City, CA, USA). The primer information is presented in Table S1.

Table S1: Primers used for qPCR

| Gene fragment knock-in (F4R4) | F:5′- TCAGGAATCTGGGTGGCATAGC -3′ |
| --- | --- |
|  | R:5′- TTGATTGGCACAATCCAAGGGT -3′ |
| Gene fragment knock-out (F4R6) | F:5′- TCAGGAATCTGGGTGGCATAGC -3′ |
|  | R:5′- TTAAGGCTGCCATGCGATCAAT -3′ |
| Wild type (F4R6) | F:5′- GACAAGTGGACTTTGGCTTCTGTT -3′ |
|  | R:5′- TTCTCCACCCCAAATGCGCTG -3′ |
| EIIa-cre | F:5′- TGGCCGCTGGAGATGACGTAGTTT -3′ |
|  | R:5′- GAACATCTTCAGGTTCTGCGGG -3′ |
