# Supplementary figures and images for: Effect of mechanical stimulation on tissue heterotopic ossification: an in vivo experimental study
Source: Front Physiol. 2023 Oct 11;14:1225898. doi: 10.3389/fphys.2023.1225898 (PMC10600381; doi:10.3389/fphys.2023.1225898)

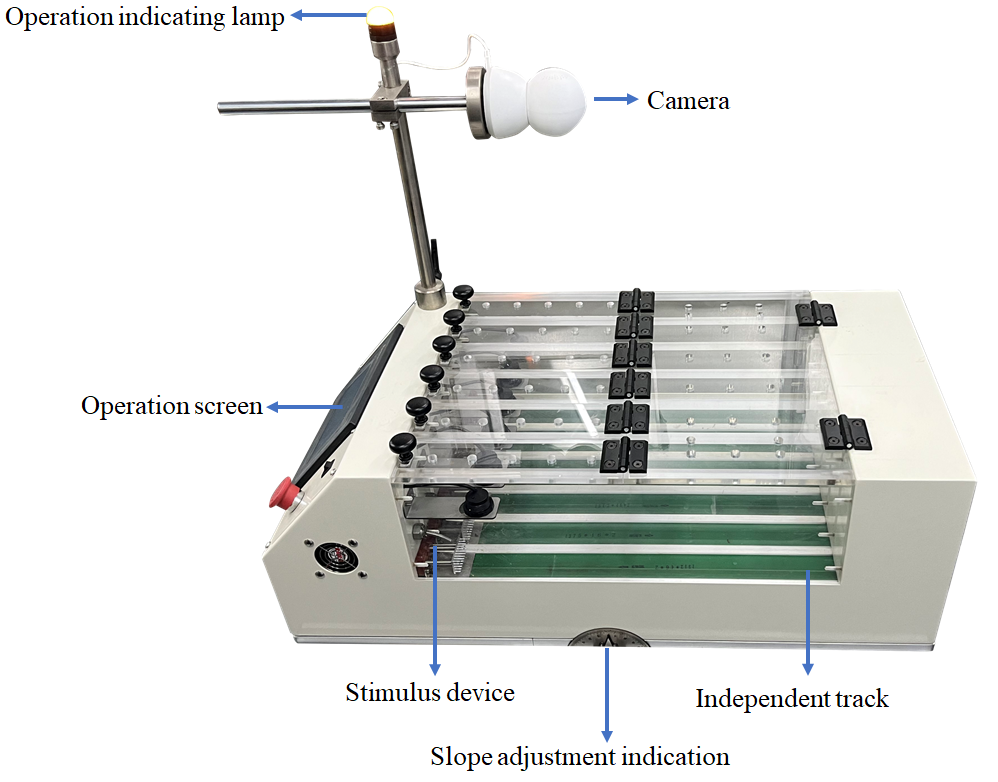

Supplement: Supplementary file 2 [file Image2.tif]

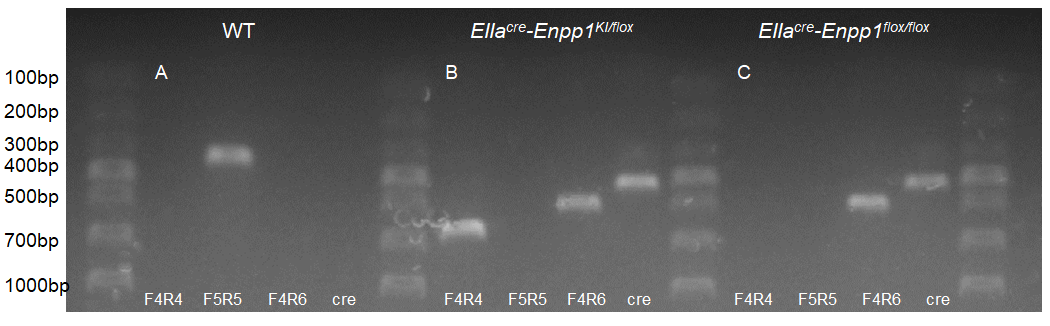

Supplement: Supplementary file 3 [file Image1.tif]
