# Supplementary material for: Effect of mechanical stimulation on tissue heterotopic ossification: an in vivo experimental study
Source: Front Physiol. 2023 Oct 11;14:1225898. doi: 10.3389/fphys.2023.1225898 (PMC10600381; doi:10.3389/fphys.2023.1225898)
Supplement: Supplementary file 4 [file Table2.DOCX]

**S2 Slope adjustable animal treadmill**

To investigate the impact of hyperactivity on the development of heterotopic ossification in genetically deficient mice, we developed a custom animal treadmill with an adjustable slope feature. This treadmill has the capacity to accommodate 6 mice simultaneously for running training. Each track can be controlled independently, allowing for individual adjustments of running parameters such as speed and time. Additionally, the slope of the treadmill can be adjusted to support inclines ranging from 0° to 90°, providing a wide range of exercise options for the mice（Fig. S2）
